# Editorial: Peptide-binding GPCRs coming of age

**DOI:** 10.3389/fendo.2023.1189508

**Published:** 2023-04-21

**Authors:** Antonella Di Pizio, Marcel Bermudez, Chris De Graaf, Ralf Jockers

**Affiliations:** ^1^ Leibniz Institute for Food Systems Biology at the Technical University of Munich, Freising, Germany; ^2^ Institute of Pharmaceutical and Medicinal Chemistry, University of Münster, Münster, Germany; ^3^ Sosei Heptares, Cambridge, United Kingdom; ^4^ Institut Cochin, Inserm, U1016, Paris, France

**Keywords:** peptides, GPCRs, drug design, drug repurposing, molecular dynamics, functional studies

G protein-coupled receptors (GPCRs) are the largest class of cell surface receptors and play a major role in the cellular perception of the environment. The A to F classification system divides GPCRs into class A (rhodopsin-like), consisting of over 80% of all GPCRs, class B (secretin-like), class C (metabotropic glutamate receptors), class D (pheromone receptors), class E (cAMP receptors), and class F (frizzled/smoothened family).

GPCRs are the targets of more than one-third of currently marketed drugs. The number of GPCRs targeted by drugs is currently ca. 16% of the GPCRs in the human genome, suggesting that the number of GPCRs to be explored for druggability is still large (1). Currently, most GPCR-targeting therapeutics are small molecules, but endogenous ligands for many GPCRs are peptides. Advances in experimental and computational approaches are fostering our mechanistic understanding of peptide-binding GPCRs and providing new opportunities to target these receptors for therapeutic indications (2, 3).

In this Research Topic, we have put together an interesting series of articles that spans different classes of peptide-binding GPCRs, providing a comprehensive picture of molecular mechanisms underlying binding and activation.

Speck et al. provided an overview of the structural information available for the angiotensin (AT) type 1 (AT1R) and type 2 (AT2R) receptors, and endothelin type B (ETBR) receptors aimed to support the molecular understanding of these important therapeutic class A peptide-binding GPCRs.

While a majority of GPCR functions have been studied in class A first, we see a constantly increasing interest in class B GPCRs, which is also reflected by the following articles.

Deganutti et al. combining supervised molecular dynamics and classic molecular dynamics simulations investigated the binding of the calcitonin gene-related peptide to the CGRP receptor. In this work, they proposed the first example of the dynamic docking of a class B GPCR. Since all the approved drugs for class B1 GPCRs are peptides that mimic the endogenous activating hormones, an understanding of agonist binding and activation is fundamental for designing novel druglike molecules in drug development campaigns.

Class B GPCRs have also been the focus of the work of Winfield et al. They explored the role of the first intracellular loop and the 8th helix in the activation of CGRP and CRF1 receptors. Mutations in different positions differentially affected three signal transduction pathways, i.e., cAMP, iCa2+, and ERK1/2. Molecular dynamics simulations were performed to investigate how the loop modulates GPCR bias.

Klenk et al. found a cleavage site in the first extracellular loop of the PTH1R receptor, a class B GPCR. Using mutagenesis, they showed that cleavage affects receptor signaling and specificity.

Langer and Latek performed structure-based virtual screening campaigns to repurpose the use of known drug molecules as blockers of VPAC1 and VPAC2 receptors, which mediate the effects of vasoactive intestinal peptide and pituitary adenylate cyclase-activating polypeptide and contribute to the regulation of intestinal motility and secretion, exocrine and endocrine secretions, and homeostasis of the immune system. They found that ticagrelor, a P2Y12 purinergic receptor antagonist, inhibits VPAC1 and VPAC2 and with molecular dynamics simulations, described its allosteric mechanism of action.

Shrivastava et al. investigated the calcium-sensing receptor (CaSR), a class C GPCR responsible for maintaining Ca2+ homeostasis in the blood, and a target of aliphatic and aromatic L-amino acids, which, as allosteric activators, increase the receptor sensitivity towards Ca2+. In pancreatic cancer, CaSR limits cell proliferation, and mutations of CaSR have been associated with Tropical Calcific Pancreatitis. Using molecular dynamics simulations, the authors characterized the molecular mechanisms by which the mutant positions affect the dynamics and stability of the protein structure.

In conclusion, this Research Topic assembles articles on a wide range of peptide-binding GPCRs with aspects as diverse as proteolytic receptor cleavage, molecular dynamics simulations, structure-based virtual ligand screening, and mutational studies on GPCR loop regions. Altogether this collection illustrates the diversity of peptide-binding GPCRs and the impact that experimentally determined receptor structures already had and will have for the future of the field.

## Author contributions

AP, MB, CG, and RJ: wrote and validated the last version of the Editorial. All authors contributed to the article and approved the submitted version.
